# Two are better than one: HPoxBS - hairpin oxidative bisulfite sequencing

**DOI:** 10.1093/nar/gky422

**Published:** 2018-06-15

**Authors:** Pascal Giehr, Charalampos Kyriakopoulos, Konstantin Lepikhov, Stefan Wallner, Verena Wolf, Jörn Walter

**Affiliations:** 1 Department of Biological Sciences, Saarland University, Campus A2.4, 66123 Saarbrücken, Saarland, Germany; 2 Computer Science Department, Saarland University, Campus E1.3, 66123 Saarbrücken, Saarland, Germany; 3 Institute for Clinical Chemistry and Laboratory Medicine, University Hospital, Franz-Josef-Strauß-Allee 11, 93053 Regensburg, Bayern, Germany

## Abstract

The controlled and stepwise oxidation of 5mC to 5hmC, 5fC and 5caC by Tet enzymes is influencing the chemical and biological properties of cytosine. Besides direct effects on gene regulation, oxidised forms influence the dynamics of demethylation and re-methylation processes. So far, no combined methods exist which allow to precisely determine the strand specific localisation of cytosine modifications along with their CpG symmetric distribution. Here we describe a comprehensive protocol combining conventional hairpin bisulfite with oxidative bisulfite sequencing (HPoxBS) to determine the strand specific distribution of 5mC and 5hmC at base resolution. We apply this method to analyse the contribution of local oxidative effects on DNA demethylation in mouse ES cells. Our method includes the HPoxBS workflow and subsequent data analysis using our developed software tools. Besides a precise estimation and display of strand specific 5mC and 5hmC levels at base resolution we apply the data to predict region specific activities of Dnmt and Tet enzymes. Our experimental and computational workflow provides a precise double strand display of 5mC and 5hmC modifications at single base resolution. Based on our data we predict region specific Tet and Dnmt enzyme efficiencies shaping the distinct locus levels and patterns of 5hmC and 5mC.

## INTRODUCTION

In mammals, DNA methylation is restricted to the C5 position of cytosine and is predominantly found in a CpG context (1–3). The precise control of its establishment and maintenance is tightly controlled by the DNA methyl-transferases Dnmt1, Dnmt3a and Dnmt3b. All three enzymes catalyse the transfer of a methyl group from s-adenosyl methionine to cytosine.

Dnmt1 is associated with the replication machinery by directly interacting with Uhrf1 and PCNA (4–7). The protein complex modulates the preferred recognition of Dnmt1 for hemimethylated CpGs, such that Dnmt1 acts as a copying enzyme for existing methylation patterns from the old to the newly synthesised DNA strand, maintaining original methylation patterns across cell divisions (8,9). This process is one of the key mechanisms of epigenetic inheritance.

On the other hand, Dnmt3a and Dnmt3b are the key enzymes to methylate CpG dinucleotides in the first place. They are called ‘*de novo*’ methyltransferases and mainly act on unmethylated DNA during epigenetic programming phases of development and differentiation (10,11). However, there are numerous indications that the strict separation of *de novo* and maintenance methylation functions between Dnmt1 and Dnmt3a/Dnmt3b is not definite. Instead, under certain conditions, these enzymes exhibit overlapping functions (12–14).

Moreover, 5-methyl cytosine (5mC) can be oxidised by a group of oxigenases called ten-eleven translocation enzymes (Tets) (15,16). Under consumption of oxygen and 2-oxo-glutarate, these Fe(II) dependent dioxigenases oxidise 5mC in a first reaction to 5-hydroxymethyl cytosine (5hmC), followed by 5-formyl cytosine (5fC) and finally 5-carboxy cytosine (5caC) (17–19). The most abundant form of these oxidised cytosine variants is 5hmC. Recent publications show that 5hmC can be found in numerous cell types such as embryonic stem cells (ESC), neurons or liver cells (20–22). The current knowledge suggests that 5hmC, like 5mC, imposes an epigenetic regulatory function through the recognition of specific reader proteins.

In zygotes 5mC is extensively converted into 5hmC mainly on the paternal (sperm derived) chromosomes (23,24). Furthermore, in subsequent cell divisions, DNA methylation decreases, suggesting that 5hmC under certain conditions promotes genome wide DNA methylation reprogramming (25,26). Based on this, and other observations, several mechanisms have been proposed how 5hmC contributes to a passive (replication dependent) and active (non-replicative) loss of DNA methylation (26–31).

In order to better understand and comprehensively follow such processes over cell divisions, accurate base resolution detection methods discriminating 5mC and 5hmC are essential.

One such method is the oxidative bisulfite conversion (oxBS). In addition to a standard bisulfite treatment, where both 5mC and 5hmC remain unconverted and indistinguishable as cytosine after sequencing, a pre-bisulfite oxidation reaction converts 5hmC to 5fC, which will be converted by bisulfite to 5f-uracil and to thymine in the subsequent PCR (32,33). By comparing the readout of standard bisulfite sequencing (BS) and oxBS, one can determine the amount of 5mC and 5hmC for each modified cytosine within the DNA.

Since bisulfite modification based methods only work efficiently on single stranded DNA, the subsequent sequencing information only covers the methylation status of one DNA strand. It is therefore impossible to deduce the symmetry of modifications at CpG dyads in double stranded (ds) DNA. To overcome this limitation, Laird *et al*. developed a method of bisulfite sequencing which physically links DNA strands by the addition of a hairpin linker, in other words a short hairpin oligo nucleotide is attached onto the DNA to prevent a physical separation of the upper (Watson) and lower (Crick) strand during bisulfite treatment (34–36).

In order to monitor the distribution of 5mC and 5hmC in ds-DNA, we here describe a protocol which combines conventional hairpin bisulfite sequencing (HPBS) with oxBS (30).

## MATERIALS AND METHODS

**Figure 1.**
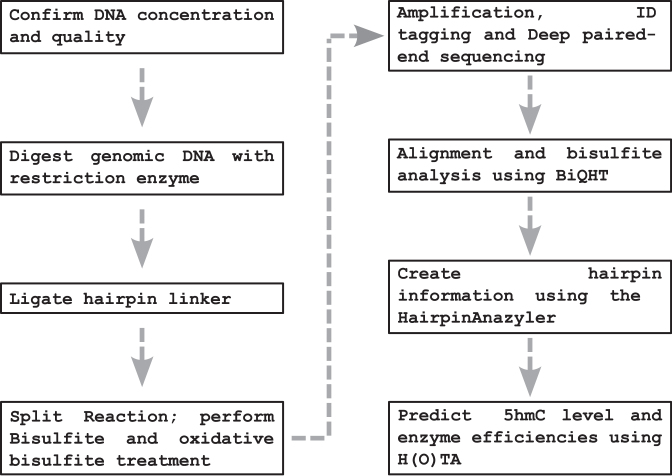
HPoxBS pipeline. Individual steps of HPoxBS starting from DNA quality assessment to 5hmC prediction and enzyme efficiency estimation.

Hairpin oxidative bisulfite sequencing (HPoxBS) comprises a series of biochemical reaction and purification steps. First, fragmented genomic DNA is ligated to a synthetic hairpin linker (37). The ligated DNA is then used for BS and oxBS treatment, sequence specific PCR amplification and finally next generation based sequencing (NGS). Figure 1 provides a general outline of the individual steps of the method.

### Digestion of genomic DNA

**Figure 2.**
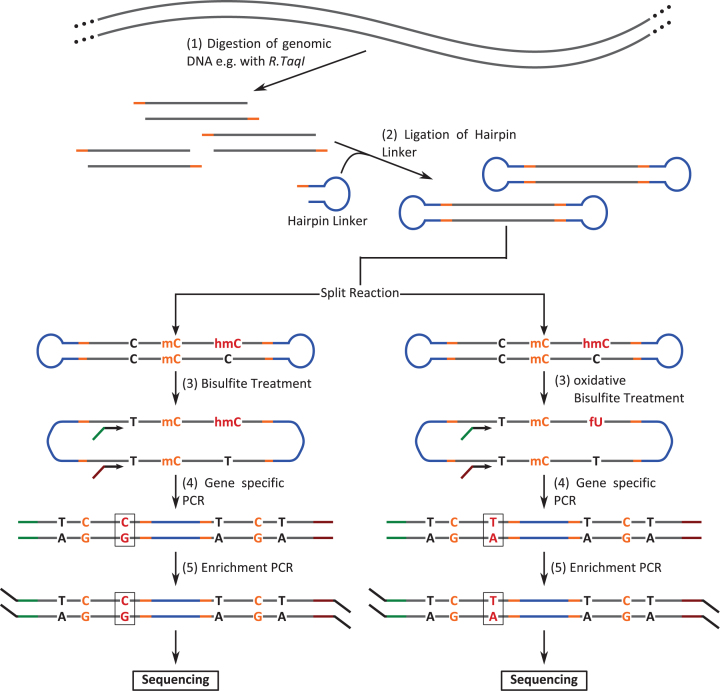
Experimental workflow of HPoxBS. (1) Genomic DNA is enzymaticaly digested; (2) DNA strands are linked covalently by ligation of a hairpin linker; (3) after ligation the reaction is split and treated with BS or oxBS; (4) region of interest is amplified and sequencing adapters are introduced; (5) multiplexed enrichment PCR including ID tagging

The first step of the experimental procedure is the digestion of genomic DNA (Figure 2). The optimal restriction enzyme (RE) used for HPoxBS should fulfill the following conditions: The restriction site should be in close proximity to the CpGs of interest, as it provides the anchor for the hairpin linker ligation. The distance between restriction site and region of interest should be ≤250 bp when using a 2× 300 bp paired sequencing mode on an Illumina MiSeq platform.

We recommend the usage of type II REs generating 3′ or 5′ overhangs to increase the ligation efficiency of the hairpin linker. Alternatively, blunt end DNA can be A-tailed using Klenow Fragment (3′ → 5′ exo-). The RE should not be sensitive against both 5mC and 5hmC, thereby avoiding a bias of the analysis by blocked restriction. Ideally, no CpG should be present within the restriction site. We have successfully used the enzymes *R.BsaWI, R.DdeI* and *R.TaqI*.

Following DNA preparation and hairpin linker ligation, the oxBS treatment includes two harsh chemical modifications, which strongly increase the risk of damaging the input DNA. It is recommended to use sufficient amounts of high quality (desalted, pure) DNA to compensate for the loss of amplifiable DNA, inevitably caused by chemical fragmentation and depurination.

We recommend to start with 300–500 ng genomic DNA digested in a buffered 20 μl reaction using a 5–10× excess of RE (units/μg). To ensure complete digestion of the DNA, the incubation should be performed overnight (12 h). After digestion is complete, the enzyme must be inactivated. We only use temperature and no chemical inactivation, as in our experience this negatively affected the ligation of the hairpin linker. The amount of DNA might be reduced in case the DNA has sufficient quality and integrity. Alternatively, when operating with very low cell numbers, HPoxBS can be applied using a ‘one tube’ reaction without prior DNA isolation. We demonstrate this application for the analysis of primordial germ cells (see supplement sections S.2 and S.3). However, if possible, we advice the use of sufficient amount of isolated high quality DNA to obtain optimal results.

### Hairpin linker design and ligation

**Figure 3.**
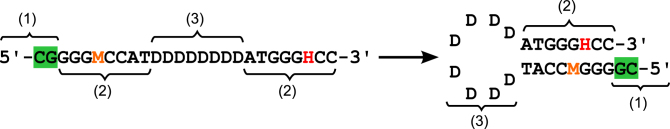
Hairpin linker structure. Example of a hairpin linker in unfolded (left) and annealed (right) conformation matching a 5′-overhang created by the restriction enzyme R.TaqI. (1) green = restriction enzyme complementary 5′-CG overhang; (2) = stem structure facilitating the folding; (3) = loop structure with unique molecular identifier sequence; M = 5mC, H = 5hmC, D = A, T or G.

The hairpin linker contains a single stranded overhang complementary to the genomic DNA overhangs generated by the RE. Figure 3 shows an example with a two base 5′-CpG overhang generated by the RE *R.TaqI*.

The hairpin linker comprises the following features: (i) A unique sequence (molecular) identifier (UMI) which allows to identify individual original ligation events and to bioinformatically remove clonal PCR amplificates from the pool of sequences (35). (ii) Unmodified cytosines at defined positions allowing to determine the overall C to T (G to A) bisulfite conversion rates. (iii) 5mC and 5hmC to deduce the rates of unwanted conversion of both modified bases due to either BS or oxBS treatment. Note that this could be expanded by including 5fC and 5caC modified bases for e.g. fCAB or MAB-Seq analysis (38–40).

Ligation of the hairpin linker, will generate closed DNA fragments (Figure 2). To minimise self-ligation of DNA fragments, hairpin linker is given in excess. The ligation reaction occurs for >4 h (or overnight) at 16°C.

### Bisulfite and oxidative bisulfite treatment

The oxBS conversion includes an oxidation step prior to the bisulfite treatment. For this oxidation (which we perform according to the manufacturer’s manual (Cambridge Epigenetix (CEGX)) the purity of the sample is of great importance as traces of salt or ethanol will cause the reaction to fail. For this reason, it is essential to purify (and desalt) the sample after the ligation reaction. We then continue with a bisulfite reaction using the TrueMethyl Kit provided by Cambridge Epigenetix (CEGX). We usually perform the following protocol:

1. After ligation, transfer the solution into a 1.5 ml reaction tube and adjust the volume to 50 μl using ddH_2_O.
2. Add 100 μl (2×) AMPure XP beads and incubate for 15 min at room temperature (RT).
3. Place the tube onto a magnetic stand and incubate for 10 min at RT.
4. Carefully discard the supernatant without disturbing the beads.
5. Keep on the magnetic stand and add 1 ml freshly prepared 80% acetonitrile and wait for 30 s. Then carefully remove and discard the supernatant.
6. Repeat the wash step from (5) three more times for a total of four wash steps.
7. Let the beads dry for 5min on the magnetic stand.
8. Without removing the tube from the magnet, add 20 μl 0.05 M NaOH.
9. Remove the reaction from the magnetic stand and resuspend the beads completely by pipetting. Incubate for 10 min at RT to elute the DNA.
10. Place the tube back onto the magnetic stand and incubate for 5 min until the suspension becomes clear.
11. Without disturbing the beads, remove 9 μl of the supernatant for BS and 9 μl for oxBS and put each into a new reaction tube. Proceed with the oxBS workflow according to Booth *et al.*

Note, for the preparation of 80% Acetonitrile and 0.05M NaOH ensure high purity of the used ddH_2_O.

**Figure 4.**
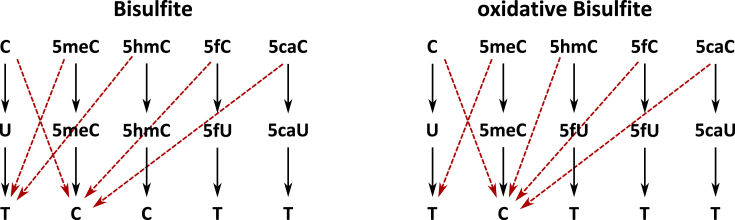
Conversion during BS and oxBS. Conversions of cytosine and its modified derivatives (upper row) during BS and oxBS (middle row) as well as their appearance after sequencing (lower row). Black straight arrows indicate the intended conversion reaction; red dashed arrows indicate possible conversion errors.

For each purified DNA we then perform two separate conversion reactions: (i) a conventional bisulfite conversion reaction and (ii) a combined oxidation and bisulfite reaction (Figure 2) and Figure 4. The single treatment with sodium bisulfite allows to simultaneously detect 5mC and 5hmC. All unmodified cytosines (as well as 5fC and 5caC, see below) are converted into uracils, while 5mC and 5hmC are not converted. In the subsequent PCR amplification and sequencing, converted cytosines will be read as thymine instead of cytosine. In the case of oxBS, 5fC will be oxidised to 5fU and converted to 5fU during bisulfite treatment. Following subsequent PCR, 5hmC will appear as thymine after sequencing. We recommend to use the TrueMethyl Kit (CEGX) to perform the bisulfite treatment. Note, when using other bisulfite protocols, ensure that the method achieves a complete conversion of 5fC and 5caC.

### Amplification of target genes

After BS and oxBS treatment, the targeted regions are amplified by PCR in which also the first part of the sequencing adapters are introduced (Table 2 and Figure 2). The PCR uses gene specific primers to amplify a specific target region ligated to the hairpin linker. For PCR we use the HOT FIREPol^®^ DNA Polymerase from Solis BioDyne, which performs well on uracil containing bisulfite templates.

**Table 1.** Typical ligation reaction for HPoxBS

20 μl	Digested chromosomal DNA (15–25 ng/μl)
2.5 μl	10 mM ATP
1.0 μl	100 pmol/μl hairpin linker
0.5 μl	400 U/μl T4 DNA ligase (New England Biolabs)
1.0 μl	ddH_2_O

**Table 2.** Typical PCR protocol for HPoxBS using HOT FIREPol^®^

**PCR protocol**	**PCR conditions**	
2.0 μl BS/oxBS hairpin sample		
3.0 μl 10× Buffer BD	95°C 15 min	
3.0 μl 25mM MgCl_2_	95°C 1 min	
2.4 μl 10mM dNTPs	X°C X min	40x
0.5 μl Forward Primer	72°C 1 min	
0.5 μl Reverse Primer	72°C 7 min	
0.7 μl HOT FIREPol^®^		
19.1 μl ddH_2_O		

After incubation, the amplified product needs to be purified to remove PCR residues, such as nucleotides, salt and primers which would interfere with downstream processes. Routinely, we perform purification using AMPure XP beads in a ratio of 1:1 (μl PCR : μl beads) or agarose gel purification using Geneaid ‘Gel/PCR DNA Fragments Kit’ following manufacturer’s instructions.

### Amplicon preparation and sequencing

Amplicon preparation for sequencing is finalised by subjecting the purified product to a second PCR (Table 3 and Figure 2). In this amplification, primers are not gene specific, but bind to the adapter part introduced during the first PCR. The second primer pair provides the adapter sequence which facilitates the binding to the sequencing platform and in addition carries a sample specific ID. The Reaction can be performed as a multiplex PCR, where several distinct amplicons can be flagged with the same ID.

**Table 3.** Multiplex-PCR protocol for sequencing preparation

**PCR protocol**	**PCR conditions**	
25.0 μl BS/oxBS hairpin sample		
5.0 μl 10× Buffer HotStarTaq	95°C 15 min	
2.0 μl 25 mM MgCl_2_	95°C 30 s	
4.0 μl 10 mM dNTPs	60°C 30 s	5×
2.5 μl Forward Primer	72°C 30 s	
2.5 μl Reverse Primer	72°C 5 min	
0.6 μl HotStarTaq^®^		
8.4 μl ddH_2_O		

In our case, subsequent sequencing is performed on an Illumina MiSeq platform using a multiplexed 2× 300 bp paired-end sequencing. For this, the products of the second PCR are again purified using AMPure XP beads with a ratio of 1:1.1 (μl PCR : μl beads). All amplicon pools are then adjusted to a concentration of 5 nM and joined for multiplexed sequencing. Following manufacture’s instructions, the pooled library is further diluted to a final concentration of 18 pM.

### Sequence alignment and methylation calling

Following demultiplexing and quality control, sequence alignment and extraction of methylation information is performed using *BiQAnalyzer HT* (BiQHT) (http://biq-analyzer-ht.bioinf.mpi-inf.mpg.de/). BiQHT is a Java based tool with graphical user interface which has been developed for locus specific DNA methylation analysis (41). The program aligns the sequencing reads against a given reference sequence and determines the methylation state for each cytosine.

To exploit all the information contained in the hairpin amplicon, four individual analysis steps have to be performed:

#### CpG methylation analysis

CpG methylation is analysed by providing a genomic reference sequence without for each locus, consisting of the unconverted DNA sequence from top and bottom strand with the converted (C replaced by T) hairpin linker sequence in between (Supplement S.8 Hairpin reference sequences). Cytosines of the hairpin linker will be analysed independently and therefore have to be replaced by Ts. The analysed methylation context has to be set to ‘CpG’. BiQHT provides several filter options to dispose unwanted sequencing reads. Routinely, we use a sequence identity of ≥0.9 for single copy genes and ≥0.8 for repetitive elements due to their sequence variability. However, filtering might be optimised for individual amplicons by including additional parameters such as conversion rate, alignment score or fraction of unrecognized sites.

#### nonCpG methylation analysis

For an unbiased detection of nonCpG methylation, all CpGs in the reference file and also in the sequencing read file must be replaced by NpNs. Furthermore, to allow nonCpG methylation detection, the analysed methylation context has to be changed to ‘C’. Usually, the filter conditions from the CpG methylation analysis can be applied.

#### Linker conversion rate

The unmodified cytosines in the hairpin linker allow the determination of cytosine conversion, unbiased by nonCpG methylation. To extract the information, sequencing reads are aligned to the genomic sequence of the hairpin linker (Supplement S.6 Linker sequences). The variable loop sequence creates UMIs which cannot be described by one reference sequence alone. Therefore, the sequencing identity filter must be reduced to ≥0.6 in order to prevent the loss of sequencing reads. Analysed methylation context has to be set to ‘C’.

#### SNP detection

BiQHT annotates single nucleotide polymorphisms (SNPs), if specified in the reference sequence. This function can also be used to determine the state of 5mC and 5hmC within the hairpin linker. Both cytosines have to be replaced by ‘N’ in the reference sequence for the analysis. BiQHT then annotates the occurring base (C or T) for both cyotsines in each read. The output can be used to calculate the conversion rate of 5mC and 5hmC during BS and oxBS (Figure 4). Applied are the same settings as in the CpG methylation analysis but in addition the option ‘output results of the SNP analysis’ must be selected.

### Restoration of double strand information

Subsequently to BiQHT, we use *Hairpinanalyzer* to restore the ds information. The *Hairpinanalyzer* is a python based script which accepts the output of BiQHT, restores the ds information and generates the following output:

1. A map of methylation pattern in form of a portable network graphic (png).
2. A text file for each sample containing the CpG methylation information of each read and in addition, position specific nonCpG methylation, conversion rates and SNPs.
3. A text summary file for all samples related to the same reference sequence.

Note that the results of BS and oxBS are stored as individual files and the level of 5hmC must be calculated by comparing both outputs. The most simple calculation is the subtraction of the mean methylation level of oxBS from BS results. Additionally, to also gain the distribution of 5hmC, this calculation has to be done for fully methylated CpGs, hemimethylated CpG on the top- and hemimethylated CpGs on the bottom strand, respectively. The script of the *Hairpinanalyzer* is available on GitHub https://github.com.

### Estimation of 5hmC and enzyme efficiency

The conversion scheme in Figure 4 suggests that 5hmC levels are simply determined by subtracting mean methylation levels of BS reactions from those of oxBS reactions. We observe that this may lead to inaccurate estimates due to random sampling of different cells and the omission of conversion errors. Therefore, we propose a more accurate approach by combining two Hidden Markov Models (HMMs), one for BS and one for oxBS, that take into account all possible conversions as outlined in Figure 4. We then link the two HMMs and calibrate the model’s parameters, such that they simultaneously fit the results of BS and oxBS. By this, we can accurately estimate 5hmC levels. For this purpose we developed H(O)TA, a MATLAB based tool which uses ds information for such calculations https://mosi.uni-saarland.de/HOTA (42). H(O)TA works with classical HPBS data, but also with data from HPoxBS experiments. Based on the information provided, H(O)TA considers the ds information and conversion rates to estimate accurate 5mC and 5hmC level as well as their ds distribution. In addition, it predicts the efficiencies of Dnmts and Tets. Furthermore, based on the ds information, H(O)TA provides a more accurate discrimination of maintenance and *de novo* methylation compared to single strand based models.

All tools come with detailed instruction for easy use. In addition, we included a test data set to the supplement information, which includes raw data from MiSeq sequencing, BiQHT and Hairpinanalyzer output as well as the input files and the results of the H(O)TA analysis.

## RESULTS

In this section, we outline the complete HPoxBS workflow (including a H(O)TA analysis) for the analysis of demethylation dynamics in mouse embryonic stem cells (ESCs). This section is followed by a brief summary of use cases on mouse primordial germ cells and human monocytes, respectively. A full description for the additional data can be found in supplement sections S.3 and S.4.

**Figure 5.**
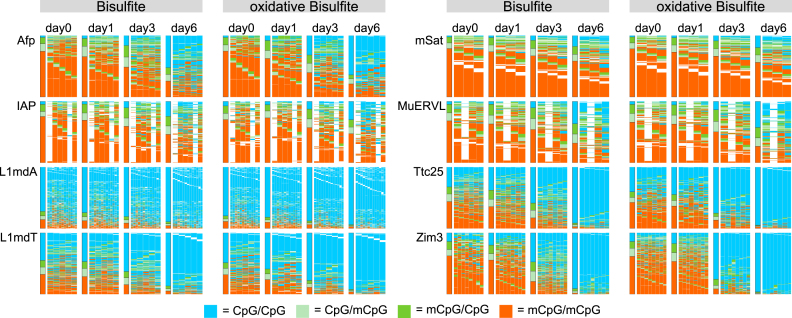
Hairpin Methylation Pattern Maps. Methylation patterns for the single copy genes Afp, Ttc25 and Zim3, as well as the retrotransposable elements IAP, L1mdT, L1mdA, mSat and MuERVL for BS and oxBS of ECS cultivated under Serum/LIF (d0) and 2i medium (d1 = 24 h 2i, d3 = 72 h 2i, d6 = 144 h 2i). Each column represents one CpG dyad, each row one sequenced chromosome. The very left column gives the mean methylation pattern over all analysed CpGs. Red = CpG dyad is modified on both DNA strands (BS = 5mC or 5hmC; oxBS = 5mC only); Dark green = CpG dyad is only modified on the plus strand (BS = 5mC or 5hmC; oxBS = 5mC only); Light green = CpG dyad is only modified on the lower strand (BS = 5mC or 5hmC; oxBS = 5mC only); Blue = CpG dyad is unmodified on both strands (BS = C only; oxBS = C or 5hmC); White = CpG dyad was not analysable.

Mouse ESCs have a high genome wide methylation status when cultivated on serum/LIF, while loosing DNA-methylation in a replication dependent manner under 2i conditional medium (43–46). We analysed ESCs under Serum/LIF (day0) conditions as well as after their transition into 2i after 24h (day1), 72h (day3) and 144h (day6). Our goal was to monitor the progressive changes in DNA-methylation and DNA-hydroxymethylation levels at three single- (Afp, Ttc25 and Zim3) and five multi copy loci (IAP, L1mdA, L1mdT, mSat and MuERVL) using HPoxBS. Following the method outlined in Figure 2 we sequenced PCR products on an Illumina MiSeq platform obtaining a mean read coverage of 5188 per locus. Figure 5 shows the CpG methylation maps for ESCs, generated after BiQHT alignment and *Hairpinanalyzer* refolding for BS and oxBS samples separately. Each column represents one CpG position and each row one unique sequence read, which corresponds to the region specific pattern of one chromosome. CpG positions modified on both DNA strands are shown in red, hemimethylated CpGs in green and unmodified CpG positions in blue. The *Hairpinanalyzer* script also generates a text file for each sample, containing the read ID, CpG methylation pattern, nonCpG Methylation and, if provided in the BiQHT reference sequence, information on SNPs.

**Figure 6.**
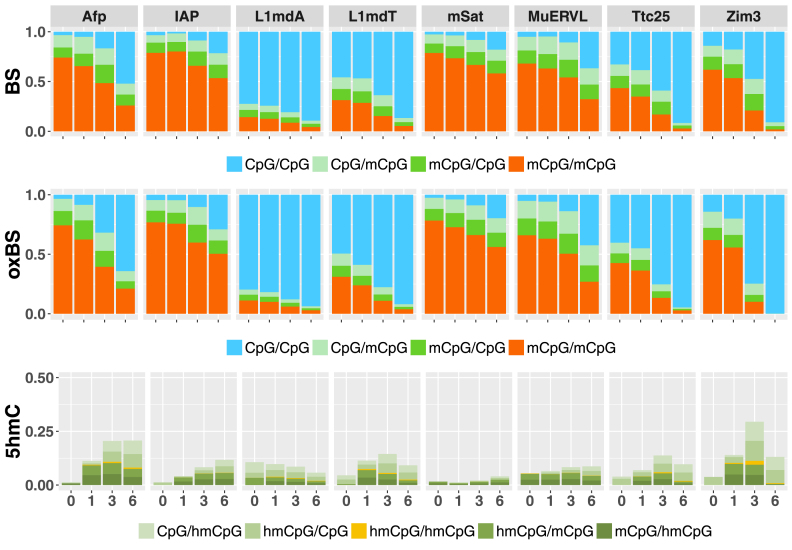
Average modification level. Mean methylation level of BS (upper panel) and oxBS (middle panel) samples as well as the predicted 5hmC amount and distribution (lower panel). x-axis = days; y-axis = 5mC/5hmC level; red = CpG dyad is modified on both DNA strands (BS = 5mC or 5hmC; oxBS = 5mC only); dark green = CpG dyad is only modified on the plus strand (BS = 5mC or 5hmC; oxBS = 5mC only); light green = CpG dyad is only modified on the lower strand(BS = 5mC or 5hmC; oxBS = 5mC only); blue = CpG dyad is unmodified on both strands (BS = C only; oxBS = C or 5hmC).

In line with our previous findings, we observe that the overall level of 5mC/5hmC decreases with region specific dynamics upon prolonged culturing of ESCs in 2i medium. This decrease occurs at retrotransposable (repetitive) elements (IAP, L1MdA, LmdT, mSat, MuERVL), as well as at single copy genes (Afp, Tct25, Zim3) (Figure 5). Using H(O)TA, we find considerable levels of 5hmC at CpG positions in most of these regions (Figure 6).

Concerning conversion quality of the oxBS reactions, we determined the conversion rate of the known C, 5mC and 5hmC positions within the hairpin linker (Figure 3). The conversion rates were calculated by dividing the number of sequenced thymines at given cytosine positions by the total number of obtained reads (Table 4 and Table 5).

\begin{eqnarray*} {{\rm Conversion}\,{\rm Rate}}&=& \frac{{\rm Number}\,{\rm of}\,{\rm T}\,{\rm at}\,{\rm C}\,{\rm Positions}}{{\rm Number}\,{\rm of}\,{\rm Reads}\,{\rm at}{\rm }\,{\rm C}\,{\rm Positions}} \end{eqnarray*}

**Table 4.** Conversion rates of C, 5mC and 5hmC of BS samples

	Afp	IAP	L1mdA	L1mdT		mSat	MuERVL	Ttc25	Zim3
C	0.996	0.999	0.995	0.993		0.996	0.993	0.994	0.995
5mC	0.0674	0.0628	0.084	0.088		0.0685	0.0819	0.0813	0.0763
5hmC	0.0765	0.0721	0.0736	0.0703		0.0642	0.0662	0.0785	0.0696

**Table 5.** Conversion rates of C, 5mC and 5hmC of oxBS samples

	Afp	IAP	L1mdA	L1mdT		mSat	MuERVL	Ttc25	Zim3
C	0.996	0.999	0.996	0.994		0.997	0.997	0.996	0.996
5mC	0.0636	0.0900	0.0795	0.0758		0.0685	0.0808	0.1078	0.0773
5hmC	0.920	0.9095	0.909	0.9323		0.93693	0.922	0.942	0.9315

Conversion of C during BS and oxBS was found to be highly efficient with a conversion rate of ≥99% (Table 4 and Table 5). However, this almost complete conversion comes at the expense of an unwanted conversion of 5mC/5hmC in the range of ∼5–10% due to the harsh bisulfite reaction conditions. Conversion of 5hmC after oxBS was 93%.

Based on the rates, individual conversion erros for BS and oxBS were calculated. A scheme of all possible conversions and conversion errors are given in Figure 4. The precise BS/oxBS values including conversion errors were then used for HMM as described in our H(O)TA tool to predict the level and distribution of 5hmC, as well as the enzyme efficiencies. H(O)TA allows to perform these predictions for individual CpGs. However, for simplicity, we here predicted the mean levels over all CpGs across one amplicon.

Figure 6 shows the mean methylation level of BS and oxBS together with the predicted 5hmC levels. The ds information demonstrates that 5hmC in most cases occurs in an asymmetric pattern paired either with C (Figure 6, lower diagram, light green) or 5mC (Figure 6, lower diagram, dark green). Only the minority of CpGs contain 5hmC in a symmetrical state (Figure 6, lower diagram, yellow).

In addition to the 5hmC distribution, H(O)TA calculates the enzyme efficiencies for Dnmts (maintenance and *de novo* methylation) and Tets (hydroxylation) for each time point. Our analysis shows that the efficiencies differ clearly between the distinct regions. In general, we can observe a loss in maintenance and *de novo* methylation activity together with an increase in hydroxylation activity. For some regions *de novo* methylation/hydroxylation efficiency is almost zero.

As a second use case we present our HPoxBS analysis of rare PGCs and nonPGC control cells, isolated from embryos at E10.5 and E11.5 of development. At this time point, PGCs are known to undergo a rapid replication dependent demethylation, probably supported by Tet mediated oxidation (47). We performed HPoxBS on repeat regions and indeed find indications for the presence of 5hmC in PGCs albeit at low levels. Our analysis demonstrates that it is possible to downscale the amount of sample material (in our case 50-80 cells/sample).

Finally, as a third application we demonstrate the analysis of human monocytes/macrophages following the dynamics of 5hmC during an ‘active’ demethylation process. In previous work we identified several deferentially methylated regions (DMRs) derived from such active demethylation and showed that the loss of 5mC is likely to be caused by Tet mediated oxidation (48). Here, we show HPoxBS results for two DMRs along a time course of 24 h (0, 12 and after 24 h) following an established differentiation protocol (49) (Supplement Section S.4). We indeed detect a region specific presence and dynamic change of 5hmC during this time course (Supplement Section 4).

## DISCUSSION

The understanding of dynamic changes of DNA methylation during development and disease is a major research area in the field of epigenetics. Such a task can only be realised if DNA modifications can be measured accurately.This is especially challenging for oxidative derivatives of 5mC considering their low abundance and unequal distribution in the genome. Furthermore there is a clear lack of reproducible and easy-to-handle assays for determination of their distribution at single base resolution. The precise knowledge however would allow to model their presumed influence on epigenetic inheritance and temporal stability. In addition, most chemical assays only allow measuring DNA methylation on one DNA strand, making it impossible to determine the precise rates of symmetric methylation and its consequences. HPoxBS is the first method to combine HPBS and oxBS for the simultaneous detection of 5mC and 5hmC levels and their distribution at both complementary DNA strands.

Our workflow not only describes the generation of sequencing data, but also bioinformatic tools applicable for data analysis and modelling. A key element in our method is the ligation of a hairpin linker to ‘fix Watson and Crick’ strands to be able to simultaneously monitor modifications in CpG dyads. In addition, we combine this approach with a double bisulfite chemistry, allowing the discrimination between 5mC and 5hmC in the DNA (32,33). We also introduce the novel concept to incorporate 5mC and 5hmC nucleotides into the hairpin linker. This allows us to directly measure their conversion rates following BS and oxBS treatment, respectively. Typically, conversion rates are determined using spike ins, i.e. small ds oligos. Such oligos are difficult to titrate and frequently perform with a different conversion efficiency. As an integrated part of the analysed DNA region, the ligated hairpin linker improves the sample specific conversion rate detection and at the same time serves as a UMI.

Demonstrating the strength of HPoxBS, we analysed the DNA methylation of several multi- and single copy sequences in embryonic stem cells (ESCs) under growth conditions in which the ESCs strongly demethylate their genome (43,45,46).

We show that HPoxBS represents a unique novel method to determine the distribution of 5mC and 5hmC as fully or hemimethylated CpG dyads. Such ds data provide a new resource for mathematic modelling of proposed DNA methylation maintenance and *de novo* methylation activities as well as active processes of DNA demethylation. More importantly, ds information allows a more accurate discrimination of maintenance and *de novo* methylation compared to singe strand data.

**Figure 7.**
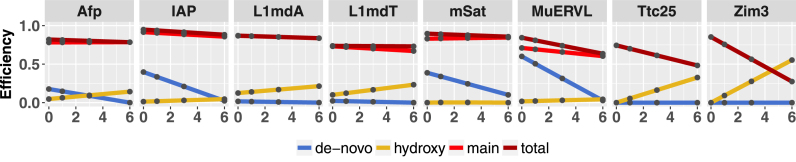
Enzyme efficiencies. Predicted enzyme efficiencies for Dnmts and Tets. Dark red = total methylation activity of Dnmts at hemimethylated CpG dyads (maintenance plus *de novo*); red = maintenance methylation of Dnmts at hemimethylated CpG dyads; blue = *de novo* activity of Dnmts at CpG dyads; yellow = hydroxylation efficiency of Tet enzymes at methylated CpG dyads. X-axis = days; Y-axis = efficiency.

Using our recently developed HMM based H(O)TA tool we predict the enzyme efficiencies in discrete regions of the genome. We observe that indeed individual loci display individual combinations of enzyme efficiencies and DNA demethylation dynamics. During the ESC culturing, i.e. the transition from serum to 2i medium, we detect a general reduction of *de novo* methylation, accompanied by an increase in hydroxylation activity (Figure 7). This observation is in concordance with the loss of Dnmt3a/3b protein and the simultaneous increase in the expression of Tet enzymes in the presence of 2i (43).

In addition, the analysis of PGCs evidences that HPoxBS can be used in experiments, where only limited amounts of cells or DNA is available, e.g. when analysing reprogramming events during early embryonic or germ cell development (Supplement S.2). Here, both active and passive demethylation processes are known to take place but the exact involvement of oxidation processes is still debated (47,49).

Rapid locus specific demethylation can also be found in somatic cells and are likewise thought to be Tet mediated. One such example is the generation of region specific demethylation during monocyte-to-macrophage maturation. Our analysis shows that indeed the active loss of 5mC clearly relies on a strong increase of 5hmC level (Supplement Section S.4).

All three examples show the broad application possibilities for HPoxBS. Moreover, these three examples demonstrate possible variations in design (DNA vs cells), molecular performance (high or low amount of material) and data analysis (and modeling).

## CONCLUSION

Taken together, we present a step by step protocol of HPoxBS which allows the detection and distribution of both 5mC and 5hmC. Overall, the outlined procedures can be modified and implemented for a number of biological questions, e.g. to understand and model the dynamic loss and gain of DNA methylation in non dividing aging cells, to characterise the heterogeneity of epihaplotypes (epigenetic chromosomal patterns) and most importantly to understand changes occurring during development and differentiation with and without DNA replication. Ultimately, in combination with new detection methods, our pipeline could easily be adjusted to likewise, describe the distribution and the behavior 5fC or 5caC (38–40,50,51).

## Supplementary Material

Supplementary Data
