# Supplementary material for: Two are better than one: HPoxBS - hairpin oxidative bisulfite sequencing
Source: Nucleic Acids Res. 2018 Jun 15;46(15):e88. doi: 10.1093/nar/gky422 (PMC6125676; doi:10.1093/nar/gky422)
Supplement: Supplementary Data [file gky422_supplemental_files.zip › supplement.HPoxBS.pdf]

Two are better than one: HPoxBS - Hairpin oxidative Bisulfite Sequencing

P. Giehr, C. Kyriakopoulos, K. Lepikhov, S. Wallner, V. Wolf, J. Walter

S.1 Hairpin oxidative Bisulfite Sequencing

500 ng of mESC DNA was cleaved with 5-10 units of restriction enzyme for 5h or overnight in a 30µl reaction atthe enzyme specific incubation temperature. For Afp, Ttc2, Zim3 the enzyme TaqI (Thermo Scientific) was used. For L1MdT, DNA was digested with BsaWI (New England Biolabs), for mSat with Eco47I (Thermo Scientific). For L1MdA and IAP, DdeI (New England Biolabs) was used. The restriction was stopped by a 20 min heat inactivation at 80°C. The restricted DNA was then subjected to ligation with T4-DNA Ligase (New England Biolabs) for 16h or overnight. We used 200 units of T4-DNA Ligase, 4µl 10mM ATP and 1µl 100µM hairpin linker. All chemicals were added directly into the restriction reaction. Finally, the volume was adjusted to 40µl using ddH_2O. BS and oxidative BS treatment was carried out using the TrueMethyl Kit from Cambridge Epigenetix (now provided by NuGEN) following manufacture's instructions. The target genes were amplified using HOTFIREPol® polymerase from Solis BioDyne. Sequencing was performed on a MiSeq Illumina system (using 2x300bp paired end sequencing). The computational analysis was done as described in the main manuscript using BiQAnalyzerHT, python script (HairpinAnalyzer) and H(O)TA.

S.2 Hairpin oxidative Bisulfite Sequencing for low cell numbers

Primordial germ cells (PGCs) were collected in 2µl M2 medium. The amount of PGCs or nonPGCs for each experiment is provided in Table 1. Cells were solubilised by adding 1µl lysis buffer (10 mM TrisHCl, 5mM EDTA), 1µl salmon sperm DNA (100 ng) and 1µl Proteinase K (1mg/ml). The reaction was incubated at 55°overnight. Proteinase K was inactivated by adding 0.7µl 8.14mM Pefabloc®SC and incubation for 1h at room temperature. Subsequently, DNA was digested in a 8µl reaction using 5U Eco47I (ThermoFisher), 0.8µl digestion buffer and 1µl 5 mM $MgCl_2$. Digestion was performed overnight at 37°C and inactivated at 65°C for 20min. The hairpin linker was ligated by adding 250U T4 DNA ligase (New England Biolabs), 1µl 10mM ATP and 0.5 µl 100µM Eco47I specific hairpin linker in an overnight reaction at 16°C. Purification, oxidation and bisulfite treatment were performed using the TrueMethyl Kit from Cambride Epigenetix. Again, amplification was performed with HOTFIREPol® polymerase from Solis BioDyne.

Table 1: Cell numbers of nonPGCs and PGCs

Sample	Number of Cells
nonPGCs	50
PGCs E10.5	60
PGCs E11.5 -1	70
PGCs E11.5 -2	80

S.3 HPoxBS on primordial germ cells

PGCs show a global loss of methylation during their development. The demethylation seems mostly driven by passive, replication dependent mechanisms, but with a possible influence of 5hmC. To test wherever HPoxBS could also be used for low cell numbers, we collected PGCs at different developmental stages (E10.5 and E11.5). We successfully generated hairpin constructs for major satellites (Figure S. 1). Applying our analysis pipeline, we find considerable levels of 5hmC at both time points (Figure S. 1). We used about 60 cells in case of nonPGCs, approximate 70 cells from E10.5 and between 80 and 90 cells for PGCs from E11.5.

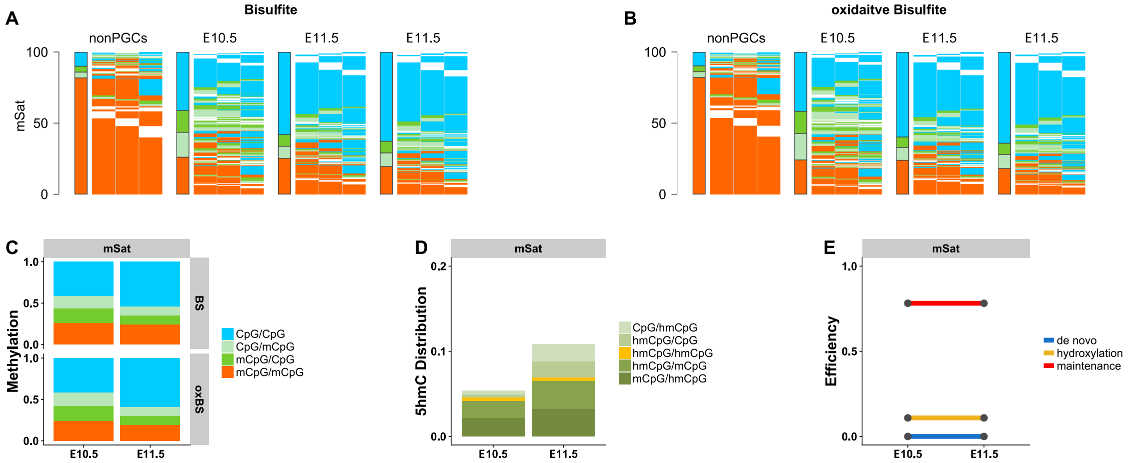

Figure S. 1: HPoxBS results for PGCs. Methylation pattern maps for BS samples, generated by the Hairpinanalyzer (A); methylation pattern maps for oxBS samples, generatd by the Hairpinanalyzer (B); average 5mC level and distribution estimated by H(O)TA (C); average 5hmC level and distribution calculated by H(O)TA (D); enzyme efficiencies for maintenance methylation, *de novo* methylation and hydroxylation predicted by H(O)TA.

S.4 Active demethylation in monocytes

The differentiation of monocytes to macrophages requires the activation of several cell type specific genes. Demethylation of the corresponding gene promoter accompany the stable activation of transcription. Interestingly, the differentiation occurs without cell division or replication which means, that the observed loss of DNA methylation corresponds to active removal of 5mC. We analysed two previously identified DMRs at the beginning (0h), in the middle (12h) and the end (24h) of monocyte-to-macrophage differentiation. Figure S.2 displays methylation pattern maps created by the Hairpinanalyser as well as the average methylation level of BS and oxBS. So far, the process of active demethylation cannot be described by H(O)TA, but will be soon implemented.

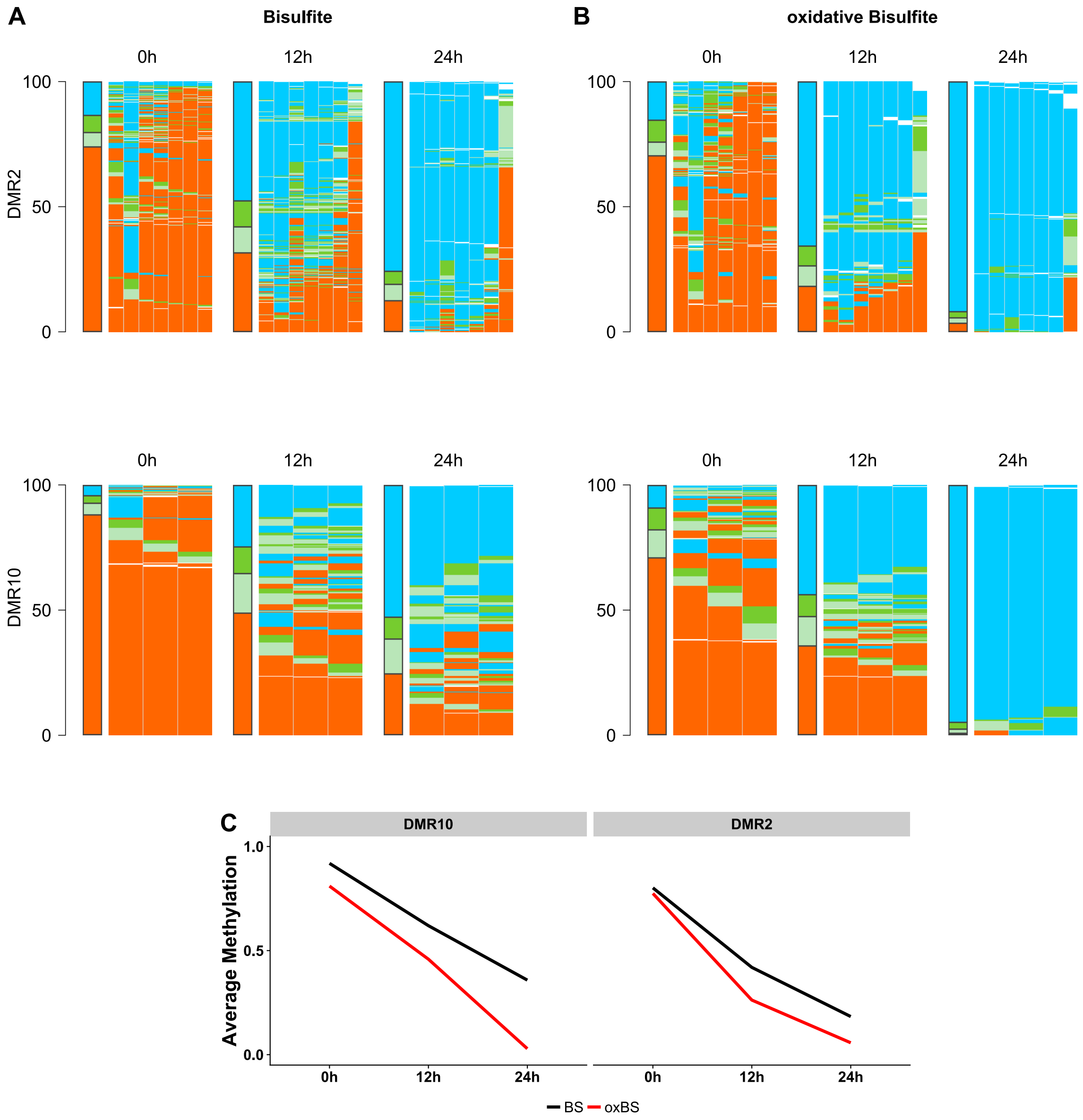

Figure S. 2: Demethylation of Monocyte DMR2 and DMR10. A: methylation pattern of hairpin BS; B: methylation pattern of hairpin oxBS; each column represents one CpG dyad, each row one sequence read i.e. one analysed DNA strand (chromosome); C: average methylation levels of DMR2 and DMR10 for BS (5mC+5hmC) and oxBS (5mC only).

S.5 BS and oxBS Data

S.5.1 BS and oxBS Data for mESCs

Table 2: Obtained reads and methylation calls for Afp

sample	# of obtained reads	# of analysed CpG	5mC/5mC	5mC/C	C/5mC	C/C
Serum (d0)-BS	8459	41898	31049	5225	4230	1394
Serum (d0)-oxBS	7200	35683	26537	3646	4300	1200
24h 2i (d1)-BS	8004	39541	25901	6693	4928	2019
24h 2i (d1)-oxBS	6732	33393	20851	4362	5363	2817
72h 2i (d3)-BS	5961	29525	14294	4879	5448	4904
72h 2i (d3)-oxBS	7163	35420	13988	5438	4736	11258
144h 2i (d6)-BS	11406	56386	14537	6203	6210	29436
144h 2i (d6)-oxBS	7024	34696	7339	2986	2177	22194

Table 3: Obtained reads and methylation calls for IAP

sample	# of obtained reads	# of analysed CpG	5mC/5mC	5mC/C	C/5mC	C/C
Serum (d0)-BS	205	874	553	148	130	43
Serum (d0)-oxBS	130	536	338	82	79	37
24h 2i (d1)-BS	254	1091	665	185	180	61
24h 2i (d1)-oxBS	144	586	368	69	107	42
72h 2i (d3)-BS	233	994	553	174	156	111
72h 2i (d3)-oxBS	159	673	367	95	109	102
144h 2i (d6)-BS	356	1561	604	259	282	416
144h 2i (d6)-oxBS	209	876	391	153	128	204

Table 4: Obtained reads and methylation calls for L1MdA

Sample	# of obtained reads	# of analysed CpG	5mC/5mC	5mC/C	C/5mC	C/C
Serum (d0)-BS	4673	55024	7833	3376	3963	39852
Serum (d0)-oxBS	3774	44457	4955	1916	2135	35451
24h 2i (d1)-BS	3334	39390	4968	2561	2571	29290
24h 2i (d1)-oxBS	3314	39237	3935	1515	1671	32116
72h 2i (d3)-BS	4528	53522	4568	2749	2869	43336
72h 2i (d3)-oxBS	3385	39909	2420	1150	1276	35063
144h 2i (d6)-BS	7033	82765	3646	2555	2697	73867
144h 2i (d6)-oxBS	4824	56647	1643	954	961	53089

Table 5: Obtained reads and methylation calls for L1MdT

Sample	# of obtained reads	# of analysed CpG	5mC/5mC	5mC/C	C/5mC	C/C
Serum (d0)-BS	6214	29632	9570	3497	3274	13291
Serum (d0)-BS	5708	27314	8720	2823	2426	13345
24h 2i (d1)-BS	8969	42648	12289	5589	4837	19933
24h 2i (d1)-oxBS	13697	64778	15498	5917	5237	38126
72h 2i (d3)-BS	7203	34411	5404	3889	3399	21719
72h 2i (d3)-oxBS	3105	14873	1678	906	774	11515
144h 2i (d6)-BS	4560	21775	1138	880	898	18859
144h 2i (d6)-oxBS	5406	25748	1000	590	515	23643

Table 6: Obtained reads and methylation calls for mSat

Sample	# of obtained reads	# of analysed CpG	5mC/5mC	5mC/C	C/5mC	C/C
Serum (d0)-BS	6845	18268	14372	1673	1732	491
Serum (d0)-oxBS	4662	12477	9776	1168	1220	313
24h 2i (d1)-BS	4618	12267	8998	1332	1492	445
24h 2i (d1)-oxBS	5489	14598	10625	1666	1741	566
72h 2i (d3)-BS	5878	15757	10513	1924	2036	1284
72h 2i (d3)-oxBS	4931	13195	8734	1597	1694	1170
144h 2i (d6)-BS	7563	20150	11724	2240	2562	3624
144h 2i (d6)-oxBS	4985	13265	7449	1613	1593	2610

Table 7: Obtained reads and methylation calls for MuERVL

Sample	# of obtained reads	# of analysed CpG	5mC/5mC	5mC/C	C/5mC	C/C
Serum (d0)-BS	767	2227	1516	293	307	111
Serum (d0)-oxBS	732	2093	1381	295	309	108
24h 2i (d1)-BS	1068	3132	1978	452	553	149
24h 2i (d1)-oxBS	1391	4131	2607	597	689	238
72h 2i (d3)-BS	847	2498	1262	420	471	345
72h 2i (d3)-oxBS	1411	4205	2276	746	735	448
144h 2i (d6)-BS	743	2186	702	321	365	798
144h 2i (d6)-oxBS	1182	3439	927	470	584	1458

Table 8: Obtained reads and methylation calls for Ttc25

Sample	# of obtained reads	# of analysed CpG	5mC/5mC	5mC/C	C/5mC	C/C
Serum (d0)-BS	8702	51478	22363	6297	5945	16873
Serum (d0)-oxBS	8180	48395	20641	3926	4338	19490
24h 2i (d1)-BS	7473	44126	15431	5340	6342	17013
24h 2i (d1)-oxBS	7679	45378	16499	4042	4448	20389
72h 2i (d3)-BS	7481	44234	7472	5705	4950	26107
72h 2i (d3)-oxBS	7669	45206	6059	2630	2501	34016
144h 2i (d6)-BS	3541	20881	595	627	538	19121
144h 2i (d6)-oxBS	7925	46621	1310	619	570	44122

Table 9: Obtained reads and methylation calls for Zim3

Sample	# of obtained reads	# of analysed CpG	5mC/5mC	5mC/C	C/5mC	C/C
Serum (d0)-BS	1574	12484	7754	1258	1695	1777
Serum (d0)-oxBS	12986	102951	63716	13448	11308	14479
24h 2i (d1)-BS	7467	59235	33002	6249	8157	11827
24h 2i (d1)-oxBS	10156	80510	43046	11222	11947	14295
72h 2i (d3)-BS	6507	51557	5202	2983	4875	38497
72h 2i (d3)-oxBS	8345	66140	13864	10965	10020	31291
144h 2i (d6)-BS	4203	33359	345	457	503	32054
144h 2i (d6)-oxBS	15675	124178	2434	4100	4761	112883

S.5.2 BS and oxBS Data for Monocytes

Table 10: Obtained reads and methylation calls for DMR2

Sample	# of obtained reads	# of analysed CpG	5mC/5mC	5mC/C	C/5mC	C/C
0h-BS	6467	44577	32911	2589	3035	6042
0h-oxBS	5423	37282	26198	2109	3249	5726
12h-BS	5710	39094	12315	4091	4070	18618
12h-oxBS	163	1115	204	90	87	734
24h-BS	4171	28480	3523	1893	1492	21572
24h-oxBS	2459	16734	556	359	441	15378

Table 11: Obtained reads and methylation calls for DMR10

Sample	# of obtained reads	# of analysed CpG	5mC/5mC	5mC/C	C/5mC	C/C
0h-BS	12612	37464	32993	1805	1077	1589
0h-oxBS	19022	56570	40167	6293	5007	5103
12h-BS	16604	49431	24057	7936	5210	12228
12h-oxBS	15623	46510	16607	5427	4019	20457
24h-BS	16330	48658	11901	6786	4264	25707
24h-oxBS	17878	53324	397	795	1470	50662

S.5.3 BS and oxBS Data for PGCs

Table 12: Obtained reads and methylation calls for mSat

Sample	# of obtained reads	# of analysed CpG	5mC/5mC	5mC/C	C/5mC	C/C
nonPGCs-BS	12401	32721	26745	1348	1397	3231
nonPGCs-oxBS	8113	21430	17603	880	884	2063
PGCs-E10.5-BS	11964	31610	8253	5556	4796	13005
PGCs-E10.5-oxBS	9824	26035	6324	4781	4092	10838
PGCs-E11.5-BS	11658	30722	7767	2635	2441	17879
PGCs-E11.5-oxBS	7828	20661	4946	1842	1527	12346
PGCs-E11.5-BS	11300	29901	5824	2916	2396	18765
PGCs-E11.5-oxBS	11362	30000	5449	2924	2426	19201

S.6 Linker Sequences

Table 13: Sequences of the used hairpin linker for Afp, IAP, L1mdA, L1mdT, mSat, MuERVL, Ttc25 and Zim3; *M* indicates 5mC, *H* 5hmC. All hairpin linker carry a 5'-phosphorylation.

Hairpin	Linker Sequnce
Afp-HP	*Pho*-CGGGG*M*CCATDDDDDDDDDATGGG*H*CC
IAP-HP	*Pho*-TNAGGG*M*CCATDDDDDDDDDATGGG*H*CC
L1mdA-HP	*Pho*-TNAGGG*M*CCATDDDDDDDDDATGGG*H*CC
L1mdT-HP	*Pho*-CCGGAGGG*M*CCATDDDDDDDDDATGGG*H*CCT
mSat-HP	*Pho*-GNCGGG*M*CCATDDDDDDDDDATGGG*H*CC
MuERVL-HP	*Pho*-GNCGGG*M*CCATDDDDDDDDDATGGG*H*CC
Ttc25-HP	*Pho*-CGGGG*M*CCATDDDDDDDDDATGGG*H*CC
Zim3-HP	*Pho*-CGGGG*M*CCATDDDDDDDDDATGGG*H*CC
DMR2-HP	*Pho*-TNAGGG*M*CCATDDDDDDDDDATGGG*H*CC
DMR10-HP	*Pho*-TNAGGG*M*CCATDDDDDDDDDATGGG*H*CC

S.7 Primer Sequences

Table 14: Primer for amplification of the analysed regions after BS or oxBS treatment.

Primer	Sequnce
Afp-HP-Forward	TTTTGTTATAGGAAAATAGTTTTTAAGTTA
Afp-HP-Reverse	AAATCACAAAACATCTTACCTATCC
IAP-HP-Forward	TTTTTTTTTTAGGAGAGTTATATTT
IAP-HP-Reverse	ATCACTCCCTAATTAACTACAAC
L1mdT-HP-Forward	TGGTAGTTTTTAGGTGGTATAGAT
L1mdT-HP-Reverse	TCAAACACTATATTACTTTAACAATTCCCA
L1mdA-HP-Forward	GTGAGTGGATTATAGTGTTTGTTTTAA
L1mdA-HP-Reverse	AAATAAATCACAATACCTACCCCAAT
mSat-HP-Forward	GGAAAATTTAGAAATGTTTAATGTAG
mSat-HP-Reverse	AACAAAAAAACTAAAAATCATAAAAA
MuERVL-HP-Forward	TAAGGGTTAGGTGGTAGTATTGAAT
MuERVL-HP-Reverse	CAAAAACCAAATAACAACATTAAAT
Ttc25-HP-Forward	TGAAAGAGAATTGATAGTTTTTAGG
Ttc25-HP-Reverse	AAAACAAAAATCTATTCCATCACTC
Zim3-HP-Forward	TTTATTTATTTGTGTGTGGTTTTTG
Zim3-HP-Reverse	CACATATCAAAATCCACTCACCTAT
DMR2-HP-Forward	TGAGTAATTGGGTTATAGGGAATAAAAAATTTT
DMR2-HP-Reverse	CTTCCTATATAAACAACTAAATCACAAAAAACA
DMR10-HP-Forward	GTTAGTATTGGTTTTGGGGTGGATTTT
DMR10-HP-Reverse	ATCTAAACTAACCTAAACCCTTACCCT

S.8 Hairpin reference sequence

TTTTGTTATAGGAAAATAGtTTTTAAGTTACAAAGCATCTTACCTATCCCAAACTCATTTTCG
TGCAATGCTTTGGACGCAGCGAAATGTAGCAGGAGGATGAGGGAAGCGGGTGTGATCCACTTC
ATGGCTGCTGGTTCCTTCACCGCAGGCAGTGCTGGAAGTGGGATGTTT**CGGGGMCCTADDDDD**
DDDTAGGGHCCCGAAACATCCCACTTCCAGCACTGCCTGCGGTGAAGGAACCAGCAGCCATGA
AGTGGATCACACCCGCTTCCCTCATCCTCCTGCTACATTTCGCTGCGTCCAAAGCATTGCACG
AAAATGAGTTTGGGATAGGTAAGATGtTTTGTGATTT

Figure S. 3: Reference Sequence for Afp

TGTCACTCCCTGATTGGCTGCAGCCCATCGGCCGAGTTGACGTCACGGGGAAGGCAGAGCACA
TGGAGTAGAGAACCACCCTCGGCATATGCGCAGATTATTTGTTTACCAC**CTAGGGMCCATNNN**
NNNNNATGGGHCCTAAGTGGTAAACAAATAATCTGCGCATATGCCGAGGGTGGTTCTCTACTC
CATGTGCTCTGCCTTCCCCGTGACGTCAACTCGGCCGATGGGCTGCAGCCAATCAGGGAGTGA
CA

Figure S. 4: Reference Sequence for IAP

TCCAATCGCGCGGAACTTGAGACTGCGGTACATAGGGAAGCAGGCTACCCGGGCCTGATCTGG
GGCACAAGTCCCTTCCGCTCGACTCGAGACTCGAGCCCCGGGCTACCTTGCCAGCAGAGTCTT
GCCCAACACCCGCAAGGGCCCACACGGGACTCCCCACGGGACC**CTNAGGGMTTATDDDDDDDD**
ATGGGHCCTNAGGGTCCCGTGGGGAGTCCCGTGTGGGCCCTTGCGGGTGTTGGGCAAGACTCT
GCTGGCAAGGTAGCCCGGGGCTCGAGTCTCGAGTCGAGCGGAAGGGACTTGTGCCCCAGATCA
GGCCCGGGTAGCCTGCTTCCCTATGTACCGCAGTCTCAAGTTCCGCGCGATTGGATTGGGGCA
GGCACTGTGATCCACTC

Figure S. 5: Reference Sequence for L1mdA

CCCGGGACCAAGATGGCGACCGCTGCTGCTGTGGCTTAGGCCGCCTCCCCAGCCGGGTGGGCA
CCTGTCCT**CCGGAGGGMCCATDDDDDDDDATGGGHCC**CCGGAGGACAGGTGCCCACCCGGCTG
GGGAGGCGGCCTAAGCCACAGCAGCAGCGGTCGCCATCTTGGTCCCGGG

Figure S. 6: Reference Sequence for L1mdT

GGAAAATTTAGAAATGTTTAATGTAGGACGTGGAATATGGCAAGAAAACTGAAAATCATGGGA
AATGAGAAACATCCACTTGTCGACTTGAAAAATGACGAAATCACTAAAAAACGTGAAAAATGA
GAAATGCACACTGAAG**GNCGGGMCCATDDDDDDDDATGGGHCC**GNCCTTCAGTGTGCATTTCT
CATTTTTCACGTTTTTTAGTGATTTCGTCATTTTTCAAGTCGACAAGTGGATGTTTCTCATTT
TTTATGATTTTTAGTTTTTTTGTT

Figure S. 7: Reference Sequence for mSat

CGCCCGAGACAAGGTGATTCTAGTTATTATAATGGACAGCGTAGACAAAAGAATGTTTATAAT
AACATACCCAGTAATGGTCAGCACAGGAGAGGTGAAATTTATAATGGCATGACTCGGTTG**GNC**
GGGMCCATDDDDDDDATGGGHCCGNTTCAACCGAGTCATGCCATTATAAATTTCACCTCTCCT
GTGCTGACCATTACTGGGTATGTTATTATAAACATTCTTTTGTCTACGCTGTCCATTATAATA
ACTAGAATCACCTTGTCTCGGGCG

Figure S. 8: Reference Sequence for MuERVL

CCAGTAGATCCTCAGCTGGGGGCAGGGATCTATTCCATCACTCCCCTTCCGTGTCGGGATTTC
GTGCAGCTCAGACGGGTCCAAGTCTTACACAAGCTGTCCTAACTGCTGTGCGTTTATATAACA
ACTACCCGGTTGTGTTTAGAAAACACTGTTTT**CGGGGMCCTADDDDDDDDATGGGHCCC**GAAA
ACAGTGTTTTCTAAACACAACCGGGTAGTTGTTATATAAACGCACAGCAGTTAGGACAGCTTG
TGTAAGACTTGGACCCGTCTGAGCTGCACGAAATCCCGACACGGAAGGGGAGTGATGGAATAG
ATCCCTGCCCC

Figure S. 9: Reference Sequence for Ttc25

CCCGGCCACCATAGTCGGATTATCCGTGGGCGGGGTGAGATGGACGGAGCGCCTTGCAGACCT
CAGGAAAACCTCCCCACGCCTGTCCGGCCTTGGCTTGGTGACAGGGAAACTGGCTGGACT**CGG**
GGMCCATDDDDDDDDATGGGHCCCGAGTCCAGCCAGTTTCCCTGTCACCAAGCCAAGGCCGGA
CAGGCGTGGGGAGGTTTTCCTGAGGTCTGCAAGGCGCTCCGTCCATCTCACCCCGCCCACGGA
TAATCCGACTATGGTGGCCGGGCAAGGACCACAC

Figure S. 10: Reference Sequence for Zim3

AGTATACACAGCGGACGTCAGCAGAGGTGGGCGGCAGGCGAGCCTCCTGCAGGAGCAGGCGGTCCCCTGAAGAAACTCCTTTC
GGAGTTGGCTCCTCCCCGACTTTTCAGGGAGGGATGTGGAGCAGACTCTGTGCCACCTGCCC**TNAGGGMTTATDDDDDDDDAT**
GGGHTTCTNAGGGCAGGTGGCACAGAGTCTGCTCCACATCCCTCCCTGAAAAGTCGGGGAGGAGCCAACTCCGAAAGGAGTTT
CTTCAGGGGACCGCCTGCTCCTGCAGGAGGCTCGCCTGCCGCCCACCTCTGCTGACGTCCGCTGTGTATACTGA

Figure S. 11: Reference Sequence for DMR2

TGTGAGGCTGTGTGGTTGCCAGGGAAGCCAGAAGAAATGACTTACTCCTGCCCCTGCCTCTAATGTCATGCGGTCACAAGTCC
CCAGAAGGTCTGGGCTGGCCTGGGCCCTTGCCCTCCCCACGGTGGGGGCTCACCCAGCCTGGGCGCGCTGGTCACAC**TNAGGG**
MCCATDDDDDDDDATGGGHCCNTGAGTGTGACCAGCGCGCCCAGGCTGGGTGAGCCCCCACCGTGGGG

Figure S. 12: Reference Sequence for DMR10

S.9 R-script Hairpinizer V2

The *Hairpinizer V2* is a small R script which builds hairpin reference sequences of a region of interest. For this, the user has to provide the genomic sequence of the region of interest, possible restriction enzymes, including their cutting sequence, as well as the sequence of the corresponding hairpin linker. *Hairpinizer V2* screens the upper strand for restriction sites, cuts the DNA, attaches the hairpin linker to the restriction site and adds the lower DNA strand sequence to the other side of the hairpin linker. Subsequently, this hairpin construct can then be used for primer design and building of reference sequences for BiQHT. The complete code of the *Hairpinizer V2* is provided on the following pages:

```
#Hairpinizer v2.0

#by Gilles Gasperoni


#v2.0 now support of degenerated restriction sites

hairpinizer<-function(seq = NULL,

                    inputID = "dna",

                    REseq = "CCGG",

                    RE.id = "MspI",

                    linker.seq = "CGGGGCCCATDDDDDDDDATGGGCCC",

                    length.hairpin.region = 300){



seq<-tolower(seq)

seq.length<-nchar(seq)

REseq<-tolower(REseq)

REseq.length<-nchar(REseq)

#Analyze REseq for degeneration...

REpatterns<-gsub("n","[a|c|g|t]",gsub("r","[a|g]",gsub("y","[c|t]",gsub("w","[a|t]",gsub("s","[c|g]",


gsub("m","[a|c]",gsub("k","[t|g]",gsub("b","[g|c|t]",gsub("h","[a|c|t]",gsub("d","[a|g|t]",gsub("v","[
a|g|c]",REseq)))))))))))



out<-
data.frame(inputID=inputID,sequenceID=NA,RE=RE.id,REseq=REseq,REpatterns=REpatterns,hp_Regi
on_Length=NA,

               sequence=NA,hp_Region_Start_Relative=NA,

               hp_Region_End_Relative=NA,hp_Sequence=NA,

               hp_Sequence_ReverseComplement=NA,linker_Sequence=NA,Comment=NA)
```

```
if(length.hairpin.region>seq.length){
  print(paste(date(),".....Hairpin Region is larger than input sequence",sep=""))
}

else{
  print(paste(date(),".....Check for presence of restriction sites...",sep=""))
  re.sites<-gregexpr(REpatterns, seq)#[[1]]

if(re.sites[[1]][1]== -1){
  print(paste(date(),".....No restriction sites '",REseq,"' present in sequence",sep=""))
  out[1,"Comment"]<-paste("No restriction sites '",REseq,"' present in sequence",sep="")
}

else{
 re.hits<-as.numeric(re.sites[[1]])
 n.re.hits<-length(re.hits)
 print(paste(date(),".....Identified ",n.re.hits," Restriction sites for '",REseq,"' in sequence",sep=""))

 for (i in 1:n.re.hits){
 print(paste(date(),".....Analyze site ",i,"...",sep=""))
  re.site.start<-re.hits[i]
  hairpin.start<-re.site.start-length.hairpin.region
  re.site.end<-re.site.start+REseq.length-1
  if(i==1){
    if(hairpin.start<1){
      hairpin.start<-1
      out[i,"Comment"]<-"RE site is close to sequence start...trimmed."
    }
```

```
  }
 if(i>1){
  pre.site.end<-re.hits[i-1]+REseq.length-1
  dist.pre.site<-re.site.start-pre.site.end
  if(dist.pre.site<length.hairpin.region){
   hairpin.start<-pre.site.end+1
   out[i,"Comment"]<-"Upstream RE site is closer than desired hp length...trimmed."
  }
 }
 out[i,"hp_Region_Start_Relative"]<-hairpin.start
 out[i,"hp_Region_End_Relative"]<-re.site.end
 out[i,"hp_Region_Length"]<-re.site.end-hairpin.start+1
 temp.seq<-substr(seq,hairpin.start,re.site.end)
 out[i,"hp_Sequence"]<-temp.seq
 out[i,"hp_Sequence_ReverseComplement"]<-reverse.complement(temp.seq)
 out[i,"sequence"]<-paste(temp.seq,linker.seq,reverse.complement(sequence = temp.seq),sep="")
 out[i,"inputID"]<-inputID
 out[i,"RE"]<-RE.id
 out[i,"REseq"]<-REseq
 out[i,"REpatterns"]<-REpatterns
 out[i,"linker_Sequence"]<-linker.seq
 out[i,"sequenceID"]<-paste(inputID,"_",out[i,"RE"],"_",out[i,"hp_Region_Start_Relative"],"_",
              out[i,"hp_Region_End_Relative"],sep="")
 }#for
}#else1
}#else2

out$linker_Sequence<-linker.seq
```

```
out$RE<-RE.id

out$REseq<-REseq

out$REpatterns<-REpatterns

print(paste(date(),".....Done.",sep=""))

return(out)

}#function


#####EXAMPLE. Not Run

#seq<-
"ctgggacggcgaggacgcgaacctgcggagccgcgggtgtctgaggagccgcagggaacccccggcctagccgcgcccgcgtgtggccgga
gctgcgggccgggactgtgtccaggacagagccacaagcttgtccccagctcagggaggtccaggggcggcagagggagcgacaggctgcga
agcccaccggtgaccacgtgtgaacccgcgtgcgcccccagctcggccactccgtgcgggtctgccctcaccgcagctccggcctgccggccctg
cctgctcccgtggtctgggatgtggccccggtgaggacccggccccatcaggcacagggtggatgtctgtggagtgaggtgtgtgtgacatattca
tgtgaccacccgtgcagcgtcacgcgcctggccctgccgatgacaagggtgtgggcctgcgtgggcatgactgtgtgtgtgacacagagtgatgt
tgctgtgacccgtggctgcactccccacatcaccggctttcacagccttccggtaaagtgctgtgttctcccttctgtgtcttcgctgggacctgggg
caagggtgggtgtggcccccacagctggagtcagcttctgtggggccttcccgagccctccccaccctggaccagaggcccagctggttggagca
ggaagtacctgggctctggggtcagggatgggaaggctgaggaggcctgcgtgagctggacctggcctgggccctcctggccgtgcctgcctggt
ggtgcaggattcctggggctgatgacagacggggtagggctggggttggcgagcctcctgccgatacctcacgtagctgacctctgactcttcccc
agccaggctggccctgggagttgccggagagtcagtggatctgcaggctgcacgctggctgttacctttgcttctgggttcccacaggggtcatgg
ttctgtggttctccagtcagggaccctagcagggccatggggcgtgacttcctggaggtgtggcctagtatggccacggcagaggatgggggaag
agaaaggccccctttgtcagcccccgggctctgaaccaagctgaagccctccccctg"

#hp<-hairpinizer(seq,REseq = "AGCT",RE.id = "AluI",length.hairpin.region = 50)


##################################################################################
#############

##################################################################################
#############

##################################################################################
#############

#reverse.complement a dna sequence


strReverse <- function(x){

  sapply(lapply(strsplit(x, NULL), rev), paste, collapse="")

}
```

```
reverse.complement<-function(sequence,reverse=TRUE,complement=TRUE){

  temp<-as.character(tolower(sequence))

  if(reverse==TRUE){

    #temp<-paste(temp[length(temp):1]) #this was a major bug!!!!

    temp<-strReverse(temp)

  }

  if(complement==TRUE){

    temp<-gsub("a","1",temp)

    temp<-gsub("c","2",temp)

    temp<-gsub("g","c",temp)

    temp<-gsub("t","a",temp)


    temp<-gsub("1","t",temp)

    temp<-gsub("2","g",temp)

  }

  out<-temp

  return(out)

}



#example

#reverse.complement("agattcggggc")


################################################################################################
##################################################################

################################################################################################
##################################################################
```

```
##############################################################################
####################################################

#Run hairpinizer for multiple REs....

hps <- data.frame(REseq = c("CCGG","AGCT","TCGA","CATG","CTGCAG","GGATCC","CTNAG"),

         RE.id = c("MspI","AluI","TaqI","Nla3","PstI","BamHI", "DdeI"),

         linker = c("CGGGGCCCATDDDDDDDDATGGGCCC",

              "GGGCCCATDDDDDDDDATGGGCCC",

              "CGGGGCCCATDDDDDDDDATGGGCCC",

              "GGGCCTAATATAGTATAGGCCCCATG",

              "GGGCCTAATATAGTATAGGCCCTGCA",

              "GATCGGGCCCATDDDDDDDDATGGGCCC",

              "TNAGGGSCCATDDDDDDDDATGGGSCC"))


seq<-
"ctgggacggcgaggacgcgaacctgcggagccgcgggtgtctgaggagccgcagggaacccccggcctagccgcgcccgcgtgtggccgga
gctgcgggccgggactgtgtccaggacagagccacaagcttgtccccagctcagggaggtccaggggcggcagagggagcgacaggctgcga
agcccaccggtgaccacgtgtgaacccgcgtgcgcccccagctcggccactccgtgcgggtctgccctcaccgcagctccggcctgccggccctg
cctgctcccgtggtctgggatgtggccccggtgaggacccggccccatcaggcacagggtggatgtctgtggagtgaggtgtgtgtgacatattca
tgtgaccacccgtgcagcgtcacgcgcctggccctgccgatgacaagggtgtgggcctgcgtgggcatgactgtgtgtgtgacacagagtgatgt
tgctgtgacccgtggctgcactccccacatcaccggctttcacagccttccggtaaagtgctgtgttctcccttctgtgtcttcgctgggacctgggg
caagggtgggtgtggcccccacagctggagtcagcttctgtggggccttcccgagccctccccaccctggaccagaggcccagctggttggagca
ggaagtacctgggctctggggtcagggatgggaaggctgaggaggcctgcgtgagctggacctggcctgggccctcctggccgtgcctgcctggt
ggtgcaggattcctggggctgatgacagacggggtagggctggggttggcgagcctcctgccgatacctcacgtagctgacctctgactcttcccc
agccaggctggccctgggagttgccggagagtcagtggatctgcaggctgcacgctggctgttacctttgcttctgggttcccacaggggtcatgg
ttctgtggttctccagtcagggaccctagcagggccatggggcgtgacttcctggaggtgtggcctagtatggccacggcagaggatgggggaag
agaaaggccccctttgtcagcccccgggctctgaaccaagctgaagccctccccctg"

hpa <- do.call("rbind",lapply(1:nrow(hps),function(x){


 hairpinizer(seq = seq,

       REseq = hps[x,"REseq"],

       RE.id = hps[x,"RE.id"],

       linker.seq = hps[x, "linker"],
```

```
            length.hairpin.region = 50)


    }

    )

    )
```
