# Supplementary material for: Two are better than one: HPoxBS - hairpin oxidative bisulfite sequencing
Source: Nucleic Acids Res. 2018 Jun 15;46(15):e88. doi: 10.1093/nar/gky422 (PMC6125676; doi:10.1093/nar/gky422)
Supplement: Supplementary Data [file gky422_supplemental_files.zip › TestDataSet_PGCs.zip!/TestDataSet_PGCs/HOTA_Results_PGCs/mSat.PGCs_CpG2.pdf]

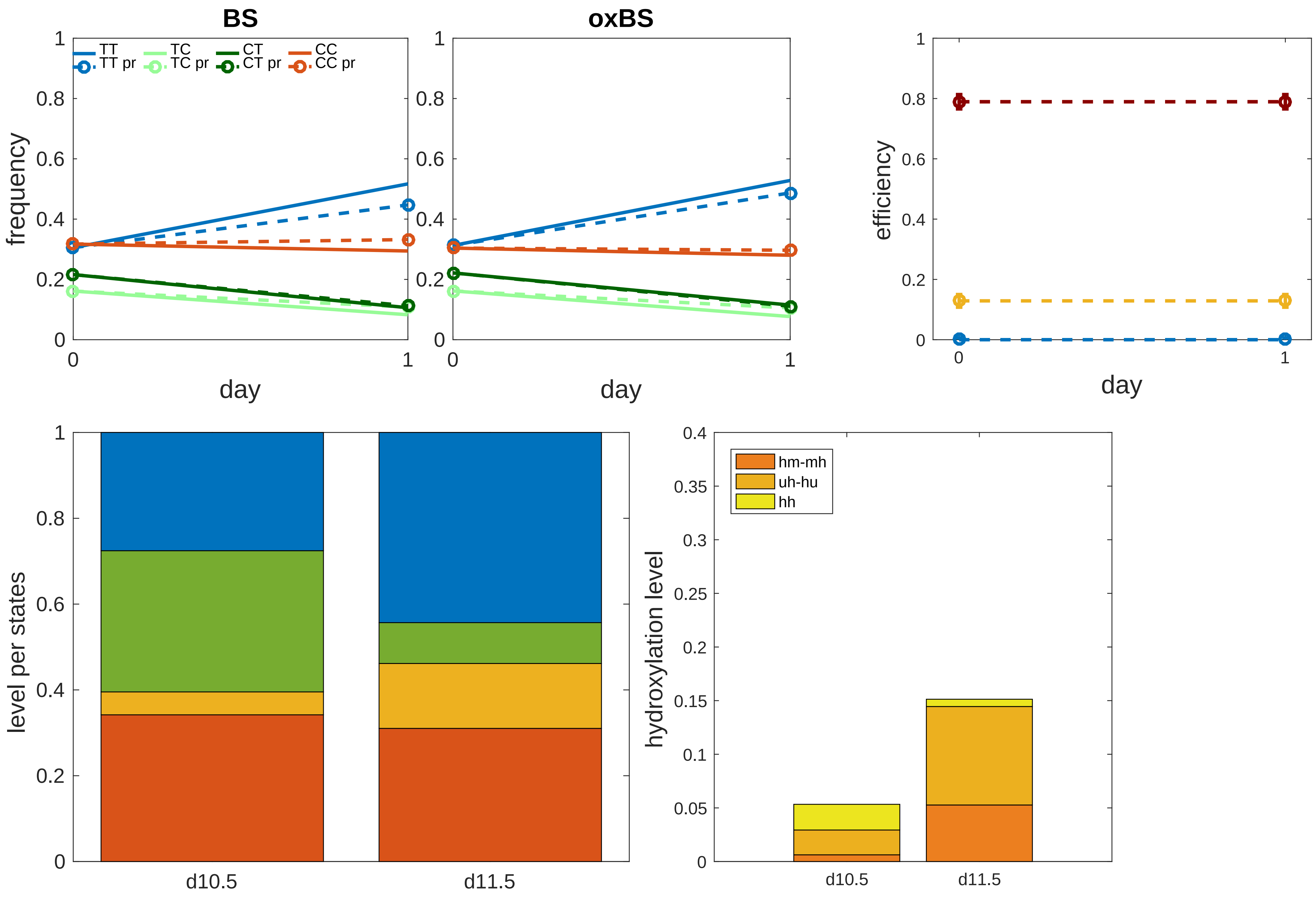
BS
oxBS
TT
TT pr
TC
TC pr
CT
CT pr
CC
CC pr
frequency
day
efficiency
level per states
d10.5
d11.5
hydroxylation level
hm-mh
uh-hu
hh
